# G+C content dominates intrinsic nucleosome occupancy

**DOI:** 10.1186/1471-2105-10-442

**Published:** 2009-12-22

**Authors:** Desiree Tillo, Timothy R Hughes

**Affiliations:** 1 Department of Molecular Genetics, University of Toronto, Toronto, ON M5S 1A8, Canada; 2 Banting and Best Department of Medical Research, University of Toronto, Toronto, ON M5S 3E1, Canada

## Abstract

**Background:**

The relative preference of nucleosomes to form on individual DNA sequences plays a major role in genome packaging. A wide variety of DNA sequence features are believed to influence nucleosome formation, including periodic dinucleotide signals, poly-A stretches and other short motifs, and sequence properties that influence DNA structure, including base content. It was recently shown by Kaplan et al. that a probabilistic model using composition of all 5-mers within a nucleosome-sized tiling window accurately predicts intrinsic nucleosome occupancy across an entire genome *in vitro*. However, the model is complicated, and it is not clear which specific DNA sequence properties are most important for intrinsic nucleosome-forming preferences.

**Results:**

We find that a simple linear combination of only 14 simple DNA sequence attributes (G+C content, two transformations of dinucleotide composition, and the frequency of eleven 4-bp sequences) explains nucleosome occupancy *in vitro *and *in vivo *in a manner comparable to the Kaplan model. G+C content and frequency of AAAA are the most important features. G+C content is dominant, alone explaining ~50% of the variation in nucleosome occupancy *in vitro*.

**Conclusions:**

Our findings provide a dramatically simplified means to predict and understand intrinsic nucleosome occupancy. G+C content may dominate because it both reduces frequency of poly-A-like stretches and correlates with many other DNA structural characteristics. Since G+C content is enriched or depleted at many types of features in diverse eukaryotic genomes, our results suggest that variation in nucleotide composition may have a widespread and direct influence on chromatin structure.

## Background

The genomes of eukaryotes are packaged into nucleosomes, comprised of approximately 147 base pairs of double-stranded DNA wrapped around an octamer of the highly conserved histone subunits[1]. Histones are the most abundant DNA binding proteins in the cell, and occupy ~80% of the yeast genome *in vivo*[2]. In the past few decades, it has become clear that the biological roles of nucleosomes extend far beyond simple DNA packaging, to include replication, DNA repair, recombination, and transcriptional regulation[3,4]. Active regulatory sequences are often depleted of nucleosomes[5-7], presumably due to steric hindrance constraints between nucleosomes and binding of most other DNA-binding proteins. The interplay between histones, DNA, and other DNA-binding proteins is therefore critical to the orchestration of transcription and other functions of the genome.

In *S. cerevisiae*, studies examining the relative incorporation of yeast genomic DNA into nucleosomes *in vitro *have demonstrated that nucleosome depletion at promoters is to a large extent programmed into the DNA sequence[8,9]. These experiments were conducted using chicken[8] or human[9] histones, which, when assembled onto yeast genomic DNA, adopted a configuration that closely resembles that of yeast nucleosomes *in vivo*. Therefore these results also indicate that the sequence preferences of nucleosomes are likely to be broadly conserved across eukarya.

To fully understand the function and evolution of gene regulation and genome packaging, it will be essential to understand the sequence preferences of nucleosomes. A variety of sequence cues have been shown to influence nucleosome sequence preference. These include nucleosome positioning[10,11] and excluding[12-15] sequences, as well as many local structural features that describe the overall deformability, curvature and flexibility of double stranded DNA[16-19] that could affect nucleosome occupancy and arrangement at particular sites in the genome. Methods to predict nucleosome positioning and occupancy from sequence have often relied on periodic dinucleotide patterns found in collections of nucleosomal sequences from both *in vivo *and *in vitro *experiments[20,21] and these patterns can explain a fraction of nucleosome positions *in vivo*[22,23]. However, analyses of sequences highly enriched in nucleosome-occupied and nucleosome-depleted regions in genome-scale and genome-wide data sets have highlighted the importance of nucleosome-excluding sequences, in particular poly-dA/dT tracts[2,8,24-27], and incorporation of these features into models of nucleosome occupancy has markedly improved prediction accuracy [2,24-26]. Some of these studies have also noted that the observed nucleosome occupancy *in vivo *correlates with and can be predicted by base composition (G+C content)[2,25,28] and other structural features of DNA [2,29], many of which, on their own, correlate with base composition. However, these observations were based on *in vivo *nucleosome occupancy, and did not directly demonstrate intrinsic nucleosome sequence preference.

Kaplan et al.[8] showed recently that a probabilistic model (hereafter referred to as the "Kaplan model") using the composition of all 5-mers within a 147-base tiling window accurately predicts nucleosome occupancy across an entire genome *in vitro*. The Kaplan model should inherently capture the effects of both base composition and aspects of large-scale structural properties which are thought to depend primarily on dinucleotide content[19]. However, the relative contributions of individual sequence features and properties are not readily apparent from the Kaplan model, which contained over 2,294 parameters. To our knowledge, there currently exists no systematic assessment of the impact of individual nucleosome excluding/attracting sequences on intrinsic nucleosome preference on a genomic scale, nor an examination of which features are redundant or dispensable in a combined model.

Here we used Lasso[30], a linear regression algorithm, to derive a greatly-simplified model for intrinsic nucleosome sequence preference. We used Lasso because: (1) Model generation is fast for large data sets (compared to other machine-learning approaches, such as SVM), (2) Lasso does subset selection, such that if given a set of highly correlated features, it will weight those that have the greatest impact, setting other feature weights to 0, thereby reducing the number of features in the final model, and (3) The end result is a simple linear equation, containing a set of easily interpreted weights for each feature. In our analysis, we obtained very similar models regardless of training/test divisions of the yeast genome, and we selected for further analysis one model that contains only 14 features and has predictive capacity nearly identical to the Kaplan model. While the 14 feature model is trained on the Kaplan *in vitro *data, it performs comparably or better than the best previous models on *in vivo *data in both yeast and *C. elegans*. The 14 feature model is heavily dependent on G+C and poly-A content, with G+C having the highest independent correlation with measured nucleosome occupancy. We suggest possible explanations and implications of the strong association between G+C content and intrinsic nucleosome occupancy.

## Results and Discussion

We first performed a feature selection step to identify which sequence features known or believed to influence nucleosome occupancy or positioning correlate with or are strongly associated with the *in vitro *nucleosome data of Kaplan et al.[8]. **Table S1 **(Additional File 1) lists the 171 features tested and the results of the tests. The features included: (a) mononucleotide frequency (i.e. G+C content); (b) predicted DNA structural characteristics (each calculated from the dinucleotide content using a simple linear formula[19]); (c) nucleosome positioning and excluding sequences from the literature[10-15]; and (d) the frequency of 4-bp sequences over a 150-bp window. We used 4-mers instead of 5-mers (as in the Kaplan model) in order to limit the number of features, and to obtain inputs that correlate independently with nucleosome occupancy (since each 4-mer occurs more frequently than nucleosomes, on average). We identified 130 features that we deemed to be associated with *in vitro *nucleosome occupancy across the yeast genome (see **Methods**), including representatives of all categories (a-d) above (**Table S1 **[Additional File 1]).

**Figure 1:**
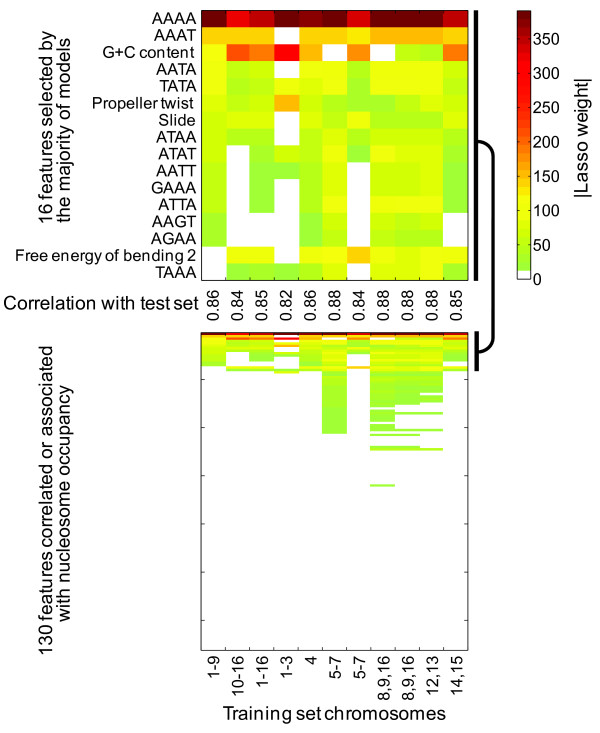
**Model feature weights selected by Lasso for eleven different training data sets**. Chromosomes from which 1,000,000 random nucleotide positions were taken are given at bottom. Correlation coefficients are given in the middle, using a test set that does not include any of the random nucleotide positions used in the training set. The top panel is a zoom-in of the 16 features that were weighted in more than half of the eleven runs. Weights do not directly reflect importance or proportion of the data that a feature explains, because features are unit-normalized prior to analysis, and can have dissimilar distributions.

We then used Lasso to learn linear models that relate these 130 features to the Kaplan et al. data. We created eleven different models, using eleven different random samples of 1,000,000 genomic positions selected from subsets of the yeast genome as training data, each with 10-fold internal cross-validation (Lasso itself chooses the number of coefficients using a cross-validation procedure within the training data). In each case, Lasso assigned nonzero weights to a similar set of features (Figures 1 and S1-3 [Additional File 1]), each of which yielded a roughly comparable correlation to test data. This result indicates that the model chosen is not strongly dependent on the subset of the data used for training. From among the models, we chose the model trained on chromosomes 1-9 for further analysis, on the basis that (a) it was an arbitrary selection, being the first model sorted numerically, and (b) it has 14 features, which is the median number among the eleven runs. Hereafter, we refer to this model as the "14 feature model", the formula for which is given in the **Methods **section.

**Figure 2:**
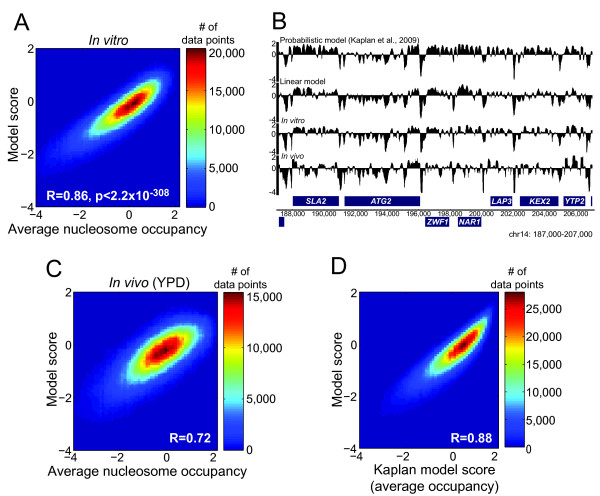
**Performance of a 14 feature linear model of intrinsic nucleosome sequence preference**. (**A**) Scatter plot vs. test set (yeast chromosomes 10-16), shown as a heat-map. Axis values are log_2 _normalized nucleosome occupancy (see **Methods**). (**B**) Model scores (probabilistic[8] and linear) and *in vivo *and *in vitro *nucleosome occupancy[8] within a 20 kb region of chromosome 14. (**C**) and (**D**) Correlation of the 14 feature model score with measured *in vivo *nucleosome occupancy in yeast (C) and with the Kaplan model across chr10-16 (test set) (D).

The 14 feature model explains a large majority of the variation in nucleosome occupancy over the yeast genome in the Kaplan et al. *in vitro *data[8] (R = 0.86 over the test set) (Figure 2A, B). This correlation is near the level of experimental reproducibility reported by Kaplan et al. (R = 0.92), and similar to that of the Kaplan model that learned 2,294 parameters (R = 0.89)[8]. We note that our models with substantially more than 14 features have correlations with the *in vitro *data as high as 0.88 (Figure 1 and S1 [Additional File 1]). The 14 feature model also correlates significantly with *in vivo *nucleosome occupancy in yeast (grown in glucose)[8] (Figure 2C) (R = 0.72, Spearman P < 2.2 × 10^-308^). The Kaplan model has a correlation coefficient of 0.74 over the same test data. Thus, the 14 feature model encapsulates the vast majority of the information in the Kaplan model. Indeed, the correlation between the 14 feature model and the Kaplan model over the entire yeast genome is 0.88 (Figure 2D).

In order to further benchmark our model, we compared the performance of the 14 feature model with published models[2,8,22-26,29,31,32] on other *in vitro *and *in vivo *nucleosome occupancy data sets, using Pearson correlation between predicted and actual data. These results are summarized in Table 1. In all cases, the 14 feature model has performance comparable to the Kaplan model and to another model (the Field model) from the same lab with a similar number of parameters as the Kaplan model[8,24]. Since the 14 feature model is trained on Illumina/Solexa sequencing data, which may have inherent biases[33], it is important to note that it also correlates well with an *in vivo *nucleosome organization from a tiling array study in yeast[2] and a sequencing-based study in *C. elegans *that was normalized using naked genomic DNA processed in the same fashion as the nucleosomal DNA[34], performing the best out of all models tested on the latter data set. Thus, our model is comparable to the Kaplan model on multiple data sets, including those generated *in vivo*, using other methods, and/or in an organism distantly related to yeast.

**Table 1:** Comparison of nucleosome occupancy prediction models on different data sets

Model	Summary	Performance (Pearson R)						Correlation with %G+C (Yeast, 150 bp windows)
		**Synthetic oligonucleotides (Microarray) **[8]	**Synthetic oligonucleotides (Sequencing) **[8]	**Yeast *in vitro ***[8]	**Yeast *in vivo ***[2]	***C. elegans *adjusted nucleosome coverage **[34]	***C. elegans *normalized occupancy **[34]	

Kaplan et al., 2009[8]	Probabilistic model based on *in vitro *5-mer preferences and periodic dinucleotide signal.	0.51*	0.45*	0.89*	**0.34**	0.47*	0.61*	0.87

Lasso model (this study)	See **Methods**.	**0.44**	**0.41**	0.86*	0.38*	0.49*	0.66*	0.85

Field et al., 2008[24]	Probabilistic model based on 5-mer preferences measured *in vivo *(yeast) and periodic dinucleotide signals.	0.47*	0.45*	**0.74**	0.39*	0.46*	0.61*	0.64

%G+C	The percentage of guanine and cytosine bases in a DNA sequence.	0.53*	0.49*	0.78*	0.25	0.42	0.47	1

Lasso model[2]	Linear regression model trained on *in vivo *nucleosome occupancy data. Uses DNA structural parameters, excluding sequences and transcription factor binding sites (ABF1, REB1, and STB2) as inputs.	0.23	0.22	**0.63**	0.45*	**0.38**	**0.5**	0.55

Peckham et al., 2007[25]	SVM classifier trained on overrepresented k-mers (k = 1-6) found in nucleosome occupied and depleted sequences determined *in vivo *yeast data.	**0.43**	**0.39**	**0.48**	0.22	0.29	0.33	0.57

Yuan and Liu, 2008[26]	Computes predicted nucleosome occupancy based on periodic dinucleotide signals found in nucleosomal and linker DNA sequences determined from *in vitro *and *in vivo *experiments in yeast	0.02	0.05	0.35	**0.27**	**0.36**	**0.48**	0.30

Miele et al., 2008[29]	Computes free energy landscape of nucleosome formation using an estimation of dinucleotide-dependent DNA flexibility and intrinsic curvature.	**0.32**	**0.26**	0.38	0.22	0.21	0.25	0.49

Segal et al., 2006[23] Downloaded January 2007	Probabilistic model trained on yeast data, using a position specific scoring matrix derived from a collection of nucleosome-bound sequences obtained from *in vitro *selection experiments.	NaN	NaN	0.05	0.09	0.05	0.05	0.07

Ioshikhes et al., 2006[22]	Computes the correlation of periodic AA/TT dinucleotide motifs in a given sequence with those found in a set of 204 eukaryotic and viral nucleosomal sequences determined through *in vivo *and *in vitro *experiments[20].	-0.03	-0.03	0.01	0.07	-0.03	-0.01	0.01

Tolstorukov et al., 2007,2008[31,32]	Estimates the dinucleotide-dependent cost of deformation caused by threading a given sequence on a template comprising the path of DNA found on the experimentally determined structure of the nucleosome core particle.	0.01	0.004	0	-0.001	-0.001	-0.001	-0.0003

Segal et al., 2006[23] Downloaded August 2009	Probabilistic model trained on yeast data, using a position specific scoring matrix derived from a collection of nucleosome-bound sequences obtained from *in vitro *selection experiments.	NaN	NaN	-0.2	0.001	-0.06	-0.05	-0.21

Pearson correlation is shown as a performance metric. Nucleosome occupancy was predicted in yeast using only sequence from the test set (chr10-16) and chromosome III in *C. elegans*. "NaN" indicates that a score of "0" was obtained for each sequence (since this model[23] requires the sequence be > 150 bp in length). Models are sorted by their average rank in performance. Asterisks (*) and text in **bold **denote the top three and top 50% performing models for each data set, respectively.

The results from this comparison also confirm that models that combine aperiodic signals perform much better at predicting nucleosome occupancy than models based primarily on periodic dinucleotide signals[22,23]. The one exception is the model of Yuan and Liu[26], which is based on periodic dinucleotide signals in nucleosomal and linker sequences identified using wavelet analysis. We note, however, that the dinucleotide features with most predictive power and the highest regression coefficients in the Yuan and Liu model have frequencies at the single base scale (i.e. have a length scale of 1)[26], suggesting that aperiodic dinucleotide composition is, perhaps unintentionally, a major component.

**Figure 3:**
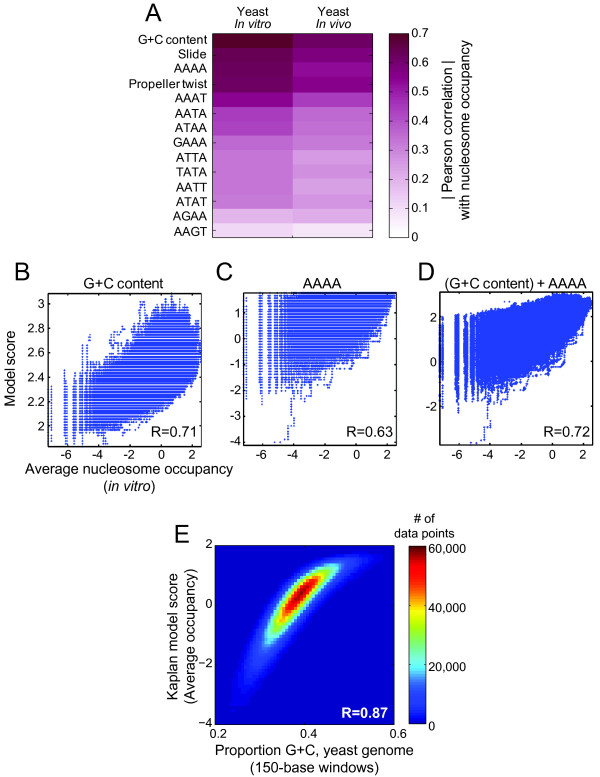
**Correlation of each of the 14 features with nucleosome occupancy**. (**A**) Graphic illustration of the correlation of each of the 14 sequence features with nucleosome occupancy in *vitr*o and *in vivo *across the yeast genome (data from Kaplan et al.[8]). (**B**-**D**) Scatter plots showing performance of linear models on test set using only G+C content (B), AAAA occurrence (C), or both (D) as inputs. (**E**) Kaplan model score vs. proportion of G+C over all 150 bp tiling windows in the yeast genome.

The most critical features in the 14 feature model are G+C content and frequency of AAAA, on the basis of two criteria. First, these two features correlate highly with nucleosome occupancy *in vitro *(R = 0.71 and 0.63, respectively), independently of all other features (Figure 3A-C). Second, a procedure in which we iteratively removed the least critical feature(s) of the model (i.e. those with the least influence on the basis of re-trained model performance after their removal) resulted in AAAA and G+C being the last two components removed (data not shown). A two-feature linear model (trained on G+C and AAAA) retained a correlation on test data of 0.72, only a marginal improvement over G+C alone (Figure 3D). From this analysis, we conclude that G+C content independently accounts for approximately half of the variation in intrinsic nucleosome occupancy (R^2 ^= 0.71^2 ^= 0.50). We note that the Kaplan model weights for individual 5-mers also scale highly with G+C content (R = 0.78, Spearman P = 3.33 × 10^-284^; data not shown) and that the scores assigned by the Kaplan model to 147-base windows across the yeast genome correlate highly with G+C content (R = 0.87, Figure 3E). Table 1 shows that other models that correlate highly with G+C content (Table 1, last column) perform well at predicting nucleosome occupancy *in vitro *and *in vivo*, and that G+C content itself is a good predictor in all data sets (Table 1): in all data sets examined, %G+C had a higher correlation that the majority of published models tested at predicting nucleosome occupancy.

We next sought to understand why these 14 features are repeatedly retained in linear models (Figure 1). Manual inspection of the components of the 14 feature model suggests a small number of overarching themes. All 11 of the 4-mers are A/T rich (eight are entirely A/T), and models of DNA structure suggest that they should retain some of the structural character of poly-A sequences (data not shown). Poly-A stretches are believed to exclude nucleosomes because they are both rigid and bent, making them less compatible with the extreme bending required for nucleosome formation, regardless of their local sequence context[14,27,35]. Sequences high in G+C will tend to lack these (and related) sequences, which may partly explain why G+C content has high overall predictive value; however, it is possible for sequences to be both G+C rich and contain small nucleosome excluding sequences, which would negatively impact nucleosome formation, explaining why a variety of poly-A-like 4-mers are retained in the model.

**Figure 4:**
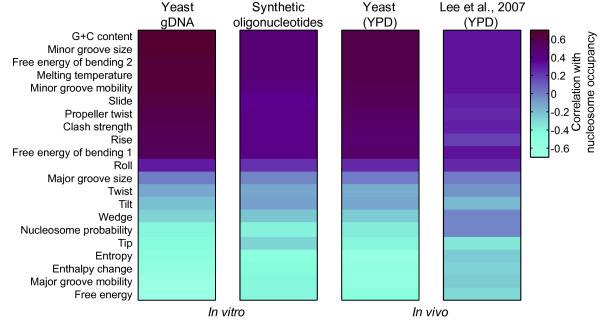
**Correlation of DNA structural parameters, calculated as the average over a 150-base window, with nucleosome occupancy *in vitro *and *in vivo***. Calculations were made using dinucleotide and other coefficients obtained from the PROPERTY database http://srs6.bionet.nsc.ru/srs6bin/cgi-bin/wgetz?-page+LibInfo+-newId+-lib+PROPERTY. Nucleosome occupancy data are from Kaplan et al.[8] and Lee et al.[2]. Pearson correlation is shown.

The importance of G+C may also be explained by the fact that this single parameter affects virtually all aspects of DNA structure. Indeed, the two overall DNA structural properties selected (propeller twist, which describes angular displacement of bases in a pair relative to each other, and slide, which describes lateral translation of base pairs relative to each other), both correlate well with G+C content when calculated as an average over a tiling window using dinucleotide tables[19] (data not shown). These and the majority of other DNA structural properties also correlate either positively or negatively with both G+C content and nucleosome occupancy *in vitro *and *in vivo *(Figure 4 and data not shown). Thus, the 14 feature model is also likely to be dominated by G+C because this parameter influences a large number of structural attributes of DNA, perhaps most critically propeller twist and slide, which may also be sufficiently important that their deviations from simple G+C content cause them to be retained in the Lasso regression. There is prior evidence for the importance of one of these features in nucleosome formation: Poly-A and related sequences are rigid and bent precisely because they are high in propeller twist, resulting in a continuous network of bifurcated hydrogen bonds[36].

**Figure 5:**
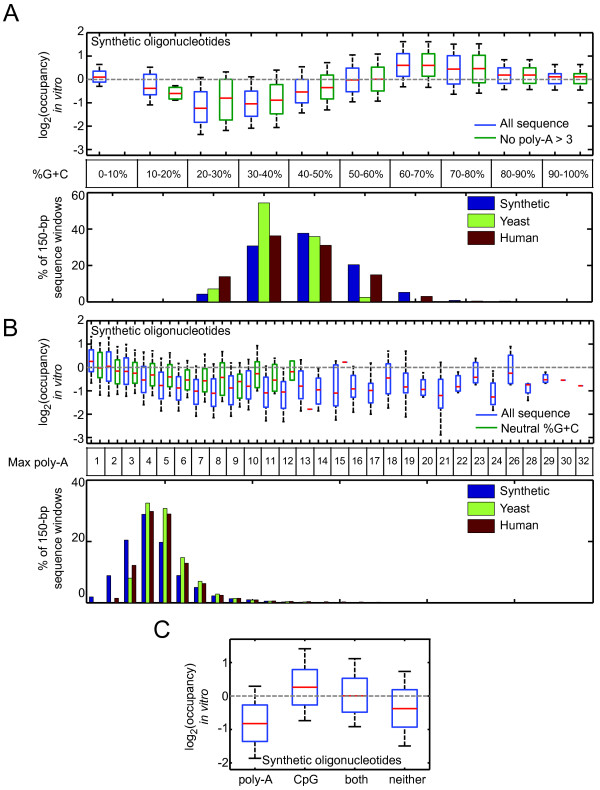
**Relative nucleosome preference of different subsets of synthetic 150-mers**. (**A**) and (**B**) Dependence of relative nucleosome preference (as log_2_(occupancy ratio)) on G+C content (A) and maximum poly-A length (B). Oligonucleotides categorized as "Neutral %G+C" in (B) are those with 45-55% G+C. Graph below shows the frequency of the selected attribute in the oligonucleotides analyzed, and also the human and yeast genomes. (**C**) Dependence of relative occupancy on poly-A content and CpG status. Poly-A containing oligonucleotides are defined as containing at least four consecutive adenine bases. CpG oligonucleotides are defined as having a G+C content ≥50%, with an observed/expected CpG ratio ≥0.6 (Obs/Exp CpG = Number of CpG * N/(num G * num C), where N = length of sequence[37]). The sequencing readout (rather than array readout) data from the Kaplan paper was used in this analysis. On all box plots, whiskers indicate 10^th ^and 90^th ^percentiles.

To gain more direct evidence for separability between G+C content and poly-A sequences as determinants of intrinsic nucleosome occupancy, we examined G+C content and poly-A sequences in an independent data set in the Kaplan et al. paper, in which nucleosomes were assembled with synthetic 150-mer sequences designed to have a broader range of unusual sequence attributes than are present in the yeast genome. Since the synthetic 150-mer nucleosome occupancy data was described by Kaplan et al. as noisier than the yeast genomic DNA occupancy data[8], due to two rounds of PCR required in the experiment, we first confirmed that the synthetic 150-mer data set displays the same global trends with respect to DNA structural parameters as does yeast genomic DNA, both *in vitro *or *in vivo *(Figure 4). We then asked whether G+C content and poly-A sequences act independently by examining the effect of one variable while holding the other within a narrow range. Figure 5A and 5B show that these parameters do act independently to a considerable degree; G+C has a major effect even if there are no poly-A tracts of length greater than three, and poly-A tracts have a clear effect even if placed in a 150-mer with neutral G+C content. We note that the behaviour at the extremes of G+C content in Figure 5A is inconsistent with the dependence of G+C shown in Figure 3B; however, there are very few data points at the extremes (Figure 5A). The *in vivo *relevance of these extremes may be very small: there are no nucleosome-sized sequence windows in yeast that are greater than 80% or less than 20% G+C, and the same is nearly true in much larger genomes (e.g. human; Figure 5A). Even human CpG islands are only 66% G+C on average. CpG-like sequences[37] among the ~27,000 oligonucleotides in this analysis[8] do have high intrinsic nucleosome occupancy overall, even if they contain poly-A sequence (Figure 5C).

Our model confirms and extends previous indications that G+C content is a major determinant of nucleosome sequence preference, demonstrating the importance of G+C content on intrinsic nucleosome occupancy. We propose that it represents a "summary feature" that both biases against poly-A-like tracts and encapsulates multiple DNA structural attributes. The 14 feature model we derive provides an extremely simple means to assess the intrinsic preference for nucleosomes to form on a given segment of DNA. Moreover, it can be used to evaluate *why *the segment has an intrinsic preference, in comparison to other sequences; the expected distribution of values for all of the model features in random sequence or across a genome is easily determined. We note that the 14 feature model does not contain any periodic component; Kaplan et al. also found that periodic signal added little to the probabilistic model[8]. We previously proposed that the predominant role of this signal may be to reinforce local translational or rotational settings[2], and we emphasize that our 14 feature model does not explicitly predict either nucleosome positioning or translational settings, nor does it account for steric effects. Nonetheless, the model scores closely mirror actual *in vitro *occupancy data obtained for the entire yeast genome, and also have strong correlations to *in vivo *nucleosome occupancy in yeast and *C. elegans *as shown in Figure 2 and Table 1 similarly or more strongly than any previous model or algorithm, and much higher than most previous approaches, particularly those that rely solely on periodic signals.

Finally, we note that G+C content as a major determinant of nucleosome occupancy has major implications for genome organisation. Our analysis indicates that in yeast simple nucleotide composition plays a direct role in nucleosome exclusion, and presumably in demarcation of promoters. Local biases in nucleotide composition have been reported in other eukaryotes, including CpG islands[37], isochores[38], and transcription start sites[39]. It will be of interest to examine how variation in base content impacts nucleosome occupancy and chromatin structure in other genomes, whether there are functional consequences, and how the intrinsic nucleosome formation signals interact with overlapping regulatory signals in the genome.

## Conclusion

We have constructed a simple predictive model of intrinsic nucleosome occupancy in which base composition (G+C content) is a major component. G+C content may be a dominant feature because it correlates with many structural properties of DNA, and also reduces the frequency of poly-A-like stretches. Since local variations in G+C content occur at many types of features in diverse eukaryotic genomes, our findings suggest that nucleotide composition may have a widespread and direct influence on chromatin structure.

## Methods

### Data sets

We converted the average nucleosome occupancy measurements from yeast (*in vitro *and *in vivo*)[8] to log_2 _scale. We also used the *in vivo *nucleosome occupancy measurements from a tiling array study in yeast[2], and measurements from an *in vivo *map of nucleosome occupancy in *C. elegans*[34] using both the "adjusted nucleosome occupancy" values (in which nucleosomal DNA was normalized with respect to micrococcal-nuclease treated genomic DNA), and raw nucleosome coverage, applying the same normalization method found in[8]. For this, we calculate a "normalized nucleosome occupancy" measure for each base pair by taking the log_2 _ratio between the basepair's total occupancy and the mean genomic average occupancy. Then, we set the genomic average to zero by subtracting the new genome-wide mean from each basepair.

### Derivation of linear model

We downloaded a MATLAB version of the Lasso algorithm[30,40]. Given a set of predictors (e.g. sequence features), and an outcome measurement (e.g. log_2 _*in vitro *nucleosome occupancy data), Lasso generates a linear model ŷ = β x_1 _+ β x_2 _+ ... β x_n_, where the output ŷ is the nucleosome occupancy prediction for a given base position, and β are the weights for each feature (x_1..n_), calculated at that position. The Lasso algorithm imposes a constraint on the sum of the weights, such that only the most important features are given non-zero weights. Input features are listed in Table 1 and were selected following[2] (but excluding transcription factor binding sequences, which are not relevant to intrinsic nucleosome sequence preferences). Briefly, for each base, we calculated the average of each structural and base composition feature in a 75-base window centered on this base; here, a 75-base window was used because it approximates the number of central basepairs (67-71 bp) bound by the histone-fold domains of the H3_2_H4_2 _tetramer of the histone octamer[1], which, in turn, dominates the free energy of histone-DNA interactions *in vitro*[41]. The frequency of sequence motifs (4-mer copy number/frequency, poly-dA/dT tract length, and nucleosome positioning and excluding sequence occurrence) was scored on both strands in 150-base windows (75 bp on the left, 74 on the right) centered on this base, because we anticipated that specific sequences would be nucleosome-excluding, and would have such an activity over the full length of the nucleosomal DNA.

For Lasso, we found that an initial reduction in feature space (to ~130 features) resulted in more stable results. We therefore selected input features as follows: for 4-mer frequency and nucleosome excluding/positioning motifs, the AUROC (area under the receiver operating curve) ≤0.45 and AUROC > 0.54. To calculate the AUROC for each sequence motif, we first sorted each 150-base sequence by *in vitro *occupancy, and used the presence or absence of the sequence feature to define positive and negative instances. For the base composition and dinucleotide feature models, we calculated the Pearson correlation to the measured *in vitro *nucleosome occupancy, and retained those with correlation > |0.10|. We then ran Lasso on the selected sequence features, training on 1,000,000 randomly selected data points from chromosomes 1-9 (or other sets of chromosomes as indicated) which had been standardized to have mean zero and unit variance (for mathematical reasons and numerical stability) and selected the optimal weights by means of 10-fold internal cross validation. The 14 feature linear model is as follows (note that these values are different from those shown in Figure 1 because we have compensated for the unit-normalization step that Lasso incorporates; **Figure S2-3 **(Additional File 1) show the equivalent of Figure 1 and S1 (Additional File 1) but with unit-normalization removed):

Intrinsic sequence preference = 1.67175 × G+C content + 0.145742 × propeller twist + 1.31928 × slide - 0.10549 × freqAAAA - 0.07628 × freqAAAT - 0.03006 × freqAAGT - 0.05055 × freqAATA - 0.02564 × freqAATT - 0.02154 × freqAGAA - 0.03949 × freqATAA - 0.02354 × freqATAT - 0.03214 × freqATTA - 0.03314 × freqGAAA - 0.0334 × freqTATA + 1.788022

Where 4-mer occurrence is calculated in 150 bp windows, and G+C content, propeller twist and slide are calculated in 75 bp windows as described above. Propeller twist and slide were calculated as averages over all dinucleotides from tables found in[19]:

Where *S(i) *is the structural feature (propeller twist, slide) score for the dinucleotide at position *i*.

For propeller twist and slide, the full equations are (from the PROPERTY database[19]http://srs6.bionet.nsc.ru/srs6bin/cgi-bin/wgetz?-page+LibInfo+-newId+-lib+PROPERTY:

Average propeller twist = (-17.3 × freqAA - 6.7 × freqAC - 14.3 × freqAG - 16.9 × freqAT - 8.6 × freqCA - 12.8 × freqCC - 11.2 × freqCG - 14.3 × freqCT - 15.1 × freqGA - 11.7 × freqGC - 12.8 × freqGG - 6.7 × freqGT - 11.1 × freqTA - 15.1 × freqTC - 8.6 × freqTG - 17.3 × freqTT)/75

Average slide = (-0.03 × freqAA - 0.13 × freqAC + 0.47 × freqAG - 0.37 × freqAT + 1.46 × freqCA + 0.6 × freqCC + 0.63 × freqCG + 0.47 × freqCT - 0.07 × freqGA + 0.29 × freqGC + 0.6 × freqGG - 0.13 × freqGT + 0.74 × freqTA - 0.07 × freqTC + 1.46 × freqTG - 0.03 × freqTT)/75

We predicted nucleosome occupancy in yeast and *C. elegans *genomes using the model scored on 150-base windows surrounding each data point in both *in vitro *and *in vivo *nucleosome maps [8,34] at 1 bp intervals.

### Comparison of nucleosome occupancy prediction models

We obtained nucleosome prediction software from the authors' website http://genie.weizmann.ac.il/software/nucleo_exe.html[8,23,24], and used the P_occ _or "average occupancy" measure. For other models[2,26,29,31,32], we requested the code from the authors. An implementation of the nucleosome positioning sequence scoring metric[22] was obtained from Dr. G.C. Yuan. Scores for the model described in[25] on all sequence data sets tested were provided by Yair Field. For all models, with the exception of the Peckham SVM[25], we predicted nucleosome occupancy across the yeast test set used for the Lasso model derived in this study (chromosomes 10-16), *C. elegans *chrIII, and synthetic 150-mer oligonucleotides (both microarray and sequencing data sets)[8], using default parameters for all models. In the case of the Peckham SVM[25], which outputs a score to every 50 bp sequence, scores over a 150-base window were calculated by averaging all contained 50 bp scores for all sequences analyzed.

## Authors' contributions

DT performed the analyses. TRH coordinated the study. TRH and DT contributed to the preparation of the manuscript.

## Supplementary Material

Additional file 1 **Supplementary data and figures**. Contains Table S1, and Figures S1-3. Click here for file

## References

[B1] Luger K Mader AW Richmond RK Sargent DF Richmond TJ Crystal structure of the nucleosome core particle at 2.8 A resolution Nature 1997 389 6648 251 260 10.1038/38444 9305837

[B2] Lee W Tillo D Bray N Morse RH Davis RW Hughes TR Nislow C A high-resolution atlas of nucleosome occupancy in yeast Nat Genet 2007 39 10 1235 1244 10.1038/ng2117 17873876

[B3] Groth A Rocha W Verreault A Almouzni G Chromatin challenges during DNA replication and repair Cell 2007 128 4 721 733 10.1016/j.cell.2007.01.030 17320509

[B4] Li B Carey M Workman JL The role of chromatin during transcription Cell 2007 128 4 707 719 10.1016/j.cell.2007.01.015 17320508

[B5] Yuan GC Liu YJ Dion MF Slack MD Wu LF Altschuler SJ Rando OJ Genome-scale identification of nucleosome positions in S. cerevisiae Science 2005 309 5734 626 630 10.1126/science.1112178 15961632

[B6] Lee CK Shibata Y Rao B Strahl BD Lieb JD Evidence for nucleosome depletion at active regulatory regions genome-wide Nat Genet 2004 36 8 900 905 10.1038/ng1400 15247917

[B7] Bernstein BE Liu CL Humphrey EL Perlstein EO Schreiber SL Global nucleosome occupancy in yeast Genome Biol 2004 5 9 R62 10.1186/gb-2004-5-9-r62 15345046 PMC522869

[B8] Kaplan N Moore IK Fondufe-Mittendorf Y Gossett AJ Tillo D Field Y LeProust EM Hughes TR Lieb JD Widom J The DNA-encoded nucleosome organization of a eukaryotic genome Nature 2009 458 7236 362 366 10.1038/nature07667 19092803 PMC2658732

[B9] Sekinger EA Moqtaderi Z Struhl K Intrinsic histone-DNA interactions and low nucleosome density are important for preferential accessibility of promoter regions in yeast Mol Cell 2005 18 6 735 748 10.1016/j.molcel.2005.05.003 15949447

[B10] Wang YH Amirhaeri S Kang S Wells RD Griffith JD Preferential nucleosome assembly at DNA triplet repeats from the myotonic dystrophy gene Science (New York, NY) 1994 265 5172 669 671 10.1126/science.8036515 8036515

[B11] Ozsolak F Song JS Liu XS Fisher DE High-throughput mapping of the chromatin structure of human promoters Nature biotechnology 2007 25 2 244 248 10.1038/nbt1279 17220878

[B12] Cao H Widlund HR Simonsson T Kubista M TGGA repeats impair nucleosome formation J Mol Biol 1998 281 2 253 260 10.1006/jmbi.1998.1925 9698546

[B13] Drew HR Travers AA DNA bending and its relation to nucleosome positioning J Mol Biol 1985 186 4 773 790 10.1016/0022-2836(85)90396-1 3912515

[B14] Suter B Schnappauf G Thoma F Poly(dA.dT) sequences exist as rigid DNA structures in nucleosome-free yeast promoters in vivo Nucleic acids research 2000 28 21 4083 4089 10.1093/nar/28.21.4083 11058103 PMC113125

[B15] Wang YH Gellibolian R Shimizu M Wells RD Griffith J Long CCG triplet repeat blocks exclude nucleosomes: a possible mechanism for the nature of fragile sites in chromosomes J Mol Biol 1996 263 4 511 516 10.1006/jmbi.1996.0593 8918933

[B16] Calladine CR Drew HR Principles of sequence-dependent flexure of DNA J Mol Biol 1986 192 4 907 918 10.1016/0022-2836(86)90036-7 3586013

[B17] Sivolob AV Khrapunov SN Translational positioning of nucleosomes on DNA: the role of sequence-dependent isotropic DNA bending stiffness J Mol Biol 1995 247 5 918 931 10.1006/jmbi.1994.0190 7723041

[B18] Brukner I Sanchez R Suck D Pongor S Sequence-dependent bending propensity of DNA as revealed by DNase I: parameters for trinucleotides The EMBO journal 1995 14 8 1812 1818 7737131 10.1002/j.1460-2075.1995.tb07169.x PMC398274

[B19] Ponomarenko JV Ponomarenko MP Frolov AS Vorobyev DG Overton GC Kolchanov NA Conformational and physicochemical DNA features specific for transcription factor binding sites Bioinformatics 1999 15 7-8 654 668 10.1093/bioinformatics/15.7.654 10487873

[B20] Ioshikhes I Bolshoy A Derenshteyn K Borodovsky M Trifonov EN Nucleosome DNA sequence pattern revealed by multiple alignment of experimentally mapped sequences Journal of molecular biology 1996 262 2 129 139 10.1006/jmbi.1996.0503 8831784

[B21] Satchwell SC Drew HR Travers AA Sequence periodicities in chicken nucleosome core DNA Journal of molecular biology 1986 191 4 659 675 10.1016/0022-2836(86)90452-3 3806678

[B22] Ioshikhes IP Albert I Zanton SJ Pugh BF Nucleosome positions predicted through comparative genomics Nat Genet 2006 38 10 1210 1215 10.1038/ng1878 16964265

[B23] Segal E Fondufe-Mittendorf Y Chen L Thastrom A Field Y Moore IK Wang JP Widom J A genomic code for nucleosome positioning Nature 2006 442 7104 772 778 10.1038/nature04979 16862119 PMC2623244

[B24] Field Y Kaplan N Fondufe-Mittendorf Y Moore IK Sharon E Lubling Y Widom J Segal E Distinct modes of regulation by chromatin encoded through nucleosome positioning signals PLoS computational biology 2008 4 11 e1000216 10.1371/journal.pcbi.1000216 18989395 PMC2570626

[B25] Peckham HE Thurman RE Fu Y Stamatoyannopoulos JA Noble WS Struhl K Weng Z Nucleosome positioning signals in genomic DNA Genome research 2007 17 8 1170 1177 10.1101/gr.6101007 17620451 PMC1933512

[B26] Yuan GC Liu JS Genomic sequence is highly predictive of local nucleosome depletion PLoS computational biology 2008 4 1 e13 10.1371/journal.pcbi.0040013 18225943 PMC2211532

[B27] Segal E Widom J Poly(dA:dT) tracts: major determinants of nucleosome organization Curr Opin Struct Biol 2009 19 1 65 71 10.1016/j.sbi.2009.01.004 19208466 PMC2673466

[B28] Schwartz S Meshorer E Ast G Chromatin organization marks exon-intron structure Nature structural & molecular biology 2009 16 9 990 995 10.1038/nsmb.1659 19684600

[B29] Miele V Vaillant C d'Aubenton-Carafa Y Thermes C Grange T DNA physical properties determine nucleosome occupancy from yeast to fly Nucleic acids research 2008 36 11 3746 3756 10.1093/nar/gkn262 18487627 PMC2441789

[B30] Tibshirani R Regression shrinkage and selection via the Lasso Journal of the Royal Statistical Society Series B-Methodological 1996 58 1 267 288

[B31] Tolstorukov MY Choudhary V Olson WK Zhurkin VB Park PJ nuScore: a web-interface for nucleosome positioning predictions Bioinformatics 2008 24 12 1456 1458 10.1093/bioinformatics/btn212 18445607 PMC3807124

[B32] Tolstorukov MY Colasanti AV McCandlish DM Olson WK Zhurkin VB A novel roll-and-slide mechanism of DNA folding in chromatin: implications for nucleosome positioning Journal of molecular biology 2007 371 3 725 738 10.1016/j.jmb.2007.05.048 17585938 PMC2000845

[B33] Dohm JC Lottaz C Borodina T Himmelbauer H Substantial biases in ultra-short read data sets from high-throughput DNA sequencing Nucleic acids research 2008 36 16 e105 10.1093/nar/gkn425 18660515 PMC2532726

[B34] Valouev A Ichikawa J Tonthat T Stuart J Ranade S Peckham H Zeng K Malek JA Costa G McKernan K A high-resolution, nucleosome position map of C. elegans reveals a lack of universal sequence-dictated positioning Genome research 2008 18 7 1051 1063 10.1101/gr.076463.108 18477713 PMC2493394

[B35] Barbic A Zimmer DP Crothers DM Structural origins of adenine-tract bending Proceedings of the National Academy of Sciences of the United States of America 2003 100 5 2369 2373 10.1073/pnas.0437877100 12586860 PMC151347

[B36] Rice PA Correll CC Protein-nucleic acid interactions: structural biology 2008 Cambridge: Royal Society of Chemistry

[B37] Gardiner-Garden M Frommer M CpG islands in vertebrate genomes J Mol Biol 1987 196 2 261 282 10.1016/0022-2836(87)90689-9 3656447

[B38] Thiery JP Macaya G Bernardi G An analysis of eukaryotic genomes by density gradient centrifugation J Mol Biol 1976 108 1 219 235 10.1016/S0022-2836(76)80104-0 826643

[B39] Aerts S Thijs G Dabrowski M Moreau Y De Moor B Comprehensive analysis of the base composition around the transcription start site in Metazoa BMC Genomics 2004 5 1 34 10.1186/1471-2164-5-34 15171795 PMC436054

[B40] Efron B Hastie T Johnstone I Tibshirani R Least Angle Regression Annals of Statistics 2004 32 2 407 499 10.1214/009053604000000067

[B41] Thastrom A Bingham LM Widom J Nucleosomal locations of dominant DNA sequence motifs for histone-DNA interactions and nucleosome positioning Journal of molecular biology 2004 338 4 695 709 10.1016/j.jmb.2004.03.032 15099738

