# Supplementary material for: G+C content dominates intrinsic nucleosome occupancy
Source: BMC Bioinformatics. 2009 Dec 22;10:442. doi: 10.1186/1471-2105-10-442 (PMC2808325; doi:10.1186/1471-2105-10-442)
Supplement: Additional file 1 — Supplementary data and figures. Contains Table S1, and Figures S1-3. [file 1471-2105-10-442-S1.PDF]

Supplementary data and figures for “G+C content dominates intrinsic nucleosome occupancy”

Desiree Tillo and Timothy R. Hughes

Table S1. Sequence features used in linear modeling. Test scores were used to determine whether each feature would be selected as an input into the model, as described in the text and **Methods**.

**Feature**	**Feature category**	**Test score (Correlation, or Area under the receiver-operating curve, AUROC)**	**Selected as model input (Y/N)?**
		**Correlation**	
GC	Base composition	0.704567	Y
Melting temperature	Dinucleotide feature	0.679502	Y
Free energy of bending 1	Dinucleotide feature[1]	0.585445	Y
Free energy of bending 2	Dinucleotide feature[1]	0.691729	Y
Clash strength	Dinucleotide feature[2]	0.586765	Y
Enthalpy	Dinucleotide feature[2]	-0.577056	Y
Entropy change	Dinucleotide feature[2]	-0.519372	Y
Free energy	Dinucleotide feature[2]	-0.662652	Y
Major mobility	Dinucleotide feature[2]	-0.603019	Y
Major size	Dinucleotide feature[2]	0.00273398	N
Minor mobility	Dinucleotide feature[2]	0.672119	Y
Minor size	Dinucleotide feature[2]	0.702452	Y
Nucleosome probability	Dinucleotide feature[2]	-0.406164	Y
Propeller twist	Dinucleotide feature[2]	0.609657	Y
Rise	Dinucleotide feature[2]	0.557754	Y
Roll	Dinucleotide feature[2]	0.322449	Y
Slide	Dinucleotide feature[2]	0.631721	Y
Tilt	Dinucleotide feature[2]	-0.182885	Y
Tip	Dinucleotide feature[2]	-0.479969	Y
Twist	Dinucleotide feature[2]	-0.136534	Y
Wedge	Dinucleotide feature[2]	-0.268244	Y
DNaseI bendability	Trinucleotide feature[3]	0.396244	Y
Satchwell nucleosome	Trinucleotide feature[4]	-0.492821	Y
		**AUROC**	
AAAA	4-mer	0.198436113	Y
AAAC	4-mer	0.422860299	Y
AAAG	4-mer	0.365546482	Y

AAAT	4-mer	0.213072102	Y
AACA	4-mer	0.489490176	N
AACC	4-mer	0.540009317	Y
AACG	4-mer	0.553974084	Y
AACT	4-mer	0.444178889	Y
AAGA	4-mer	0.419486298	Y
AAGC	4-mer	0.545520251	Y
AAGG	4-mer	0.52772231	N
AAGT	4-mer	0.426401294	Y
AATA	4-mer	0.317471279	Y
AATC	4-mer	0.457836931	N
AATG	4-mer	0.48100563	N
AATT	4-mer	0.339585398	Y
ACAA	4-mer	0.476345745	N
ACAC	4-mer	0.562976466	Y
ACAG	4-mer	0.569143219	Y
ACAT	4-mer	0.490135275	N
ACCA	4-mer	0.58607229	Y
ACCC	4-mer	0.576871247	Y
ACCG	4-mer	0.594529165	Y
ACCT	4-mer	0.551582074	Y
ACGA	4-mer	0.542541896	Y
ACGC	4-mer	0.588915324	Y
ACGG	4-mer	0.590706334	Y
ACGT	4-mer	0.555154462	Y
ACTA	4-mer	0.477989771	N
ACTC	4-mer	0.529501981	N
ACTG	4-mer	0.559383684	Y
AGAA	4-mer	0.399631803	Y
AGAC	4-mer	0.559541663	Y
AGAG	4-mer	0.538139616	N
AGAT	4-mer	0.480726412	N
AGCA	4-mer	0.588634357	Y
AGCC	4-mer	0.589509242	Y
AGCG	4-mer	0.591676673	Y
AGCT	4-mer	0.547333168	Y
AGGA	4-mer	0.528349391	N
AGGC	4-mer	0.589475141	Y
AGGG	4-mer	0.566163339	Y
AGTA	4-mer	0.47242623	N
AGTC	4-mer	0.538644871	N
AGTG	4-mer	0.545708209	Y
ATAA	4-mer	0.327365148	Y

ATAC	4-mer	0.477195733	N
ATAG	4-mer	0.472111099	N
ATAT	4-mer	0.379587737	Y
ATCA	4-mer	0.490068249	N
ATCC	4-mer	0.546225509	Y
ATCG	4-mer	0.539514014	N
ATGA	4-mer	0.472692502	N
ATGC	4-mer	0.562965212	Y
ATGG	4-mer	0.573116637	Y
ATTA	4-mer	0.368272714	Y
ATTC	4-mer	0.441961626	Y
ATTG	4-mer	0.491394518	N
CAAA	4-mer	0.394386946	Y
CAAC	4-mer	0.571520239	Y
CAAG	4-mer	0.558670514	Y
CACA	4-mer	0.559059283	Y
CACC	4-mer	0.617233369	Y
CACG	4-mer	0.58849275	Y
CAGA	4-mer	0.550248233	Y
CAGC	4-mer	0.620950948	Y
CAGG	4-mer	0.595279595	Y
CATA	4-mer	0.47159919	N
CATC	4-mer	0.578118109	Y
CATG	4-mer	0.555984603	Y
CCAA	4-mer	0.549165309	Y
CCAC	4-mer	0.615093042	Y
CCAG	4-mer	0.605246927	Y
CCCA	4-mer	0.588813892	Y
CCCC	4-mer	0.581556983	Y
CCCG	4-mer	0.578237802	Y
CCGA	4-mer	0.541689241	Y
CCGC	4-mer	0.601701629	Y
CCGG	4-mer	0.581945948	Y
CCTA	4-mer	0.530817492	N
CCTC	4-mer	0.574169339	Y
CGAA	4-mer	0.492109556	N
CGAC	4-mer	0.576399126	Y
CGAG	4-mer	0.548163868	Y
CGCA	4-mer	0.577490161	Y
CGCC	4-mer	0.600045485	Y
CGCG	4-mer	0.560006768	Y
CGGA	4-mer	0.548600085	Y
CGGC	4-mer	0.597536233	Y

CGTA	4-mer	0.548443432	Y
CGTC	4-mer	0.592736006	Y
CTAA	4-mer	0.469612204	N
CTAC	4-mer	0.56871611	Y
CTAG	4-mer	0.545530721	Y
CTCA	4-mer	0.527578849	N
CTCC	4-mer	0.578617583	Y
CTGA	4-mer	0.532021074	N
CTGC	4-mer	0.614951993	Y
CTTA	4-mer	0.467487925	N
CTTC	4-mer	0.526114952	N
GAAA	4-mer	0.299508483	Y
GAAC	4-mer	0.538543667	N
GACA	4-mer	0.556179309	Y
GACC	4-mer	0.588994859	Y
GAGA	4-mer	0.5082211	N
GAGC	4-mer	0.586514448	Y
GATA	4-mer	0.468285363	N
GATC	4-mer	0.54018811	Y
GCAA	4-mer	0.553614806	Y
GCAC	4-mer	0.603294781	Y
GCCA	4-mer	0.60220557	Y
GCCC	4-mer	0.604259933	Y
GCGA	4-mer	0.555414719	Y
GCGC	4-mer	0.599039434	Y
GCTA	4-mer	0.570867495	Y
GGAA	4-mer	0.494978792	N
GGAC	4-mer	0.587811147	Y
GGCA	4-mer	0.597122351	Y
GGCC	4-mer	0.594659104	Y
GGGA	4-mer	0.555146463	Y
GGTA	4-mer	0.558723509	Y
GTAA	4-mer	0.461211668	N
GTAC	4-mer	0.551474893	Y
GTCA	4-mer	0.55228892	Y
GTGA	4-mer	0.537592725	N
GTTA	4-mer	0.488216469	N
TAAA	4-mer	0.292303662	Y
TACA	4-mer	0.482486163	N
TAGA	4-mer	0.476456619	N
TATA	4-mer	0.386446762	Y
TCAA	4-mer	0.440612669	Y
TCCA	4-mer	0.554588782	Y

TCGA	4-mer	0.499478037	N
TGAA	4-mer	0.411896482	Y
TGCA	4-mer	0.550684515	Y
TTAA	4-mer	0.382404384	Y
CTGCTG	Peak-enriched [5]	0.630790614	Y
GCGCGC	Trough-enriched[5]	0.582412858	Y
TATAAA	Trough-enriched [5]	0.334113775	Y
TATATA	Trough-enriched [5]	0.315768853	Y
TTCGA	Trough-enriched [5]	0.473710543	N
$(GNNGNNGNN)_n$	Peak-enriched (AlignACE) [6]	0.717547834	Y
dA/dT [4,150]	Nucleosome-excluding [7]	0.198436113	Y
dG/dC [4,150]	Nucleosome-excluding [8]	0.581556983	Y
$(CTG)_n$	Nucleosome-positioning [9]	0.742392382	Y
$(GT)_5$	Peak-enriched (AlignACE) [6]	0.46273306	N
$(TGGA)_n$	Nucleosome-excluding [10]	0.543828287	Y
$(CCGNN)_n$	Nucleosome-excluding [11]	0.620116865	Y

References

1. Sivolob AV, Khrapunov SN: **Translational positioning of nucleosomes on DNA: the role of sequence-dependent isotropic DNA bending stiffness**. *J Mol Biol* 1995, **247**(5):918-931.
2. Ponomarenko JV, Ponomarenko MP, Frolov AS, Vorobyev DG, Overton GC, Kolchanov NA: **Conformational and physicochemical DNA features specific for transcription factor binding sites**. *Bioinformatics* 1999, **15**(7-8):654-668.
3. Brukner I, Sanchez R, Suck D, Pongor S: **Sequence-dependent bending propensity of DNA as revealed by DNase I: parameters for trinucleotides**. *The EMBO journal* 1995, **14**(8):1812-1818.
4. Satchwell SC, Drew HR, Travers AA: **Sequence periodicities in chicken nucleosome core DNA**. *J Mol Biol* 1986, **191**(4):659-675.
5. Ozsolak F, Song JS, Liu XS, Fisher DE: **High-throughput mapping of the chromatin structure of human promoters**. *Nature biotechnology* 2007, **25**(2):244-248.
6. Lee W, Tillo D, Bray N, Morse RH, Davis RW, Hughes TR, Nislow C: **A high-resolution atlas of nucleosome occupancy in yeast**. *Nat Genet* 2007, **39**(10):1235-1244.
7. Suter B, Schnappauf G, Thoma F: **Poly(dA.dT) sequences exist as rigid DNA structures in nucleosome-free yeast promoters in vivo**. *Nucleic acids research* 2000, **28**(21):4083-4089.
8. Drew HR, Travers AA: **DNA bending and its relation to nucleosome positioning**. *J Mol Biol* 1985, **186**(4):773-790.
9. Wang YH, Amirhaeri S, Kang S, Wells RD, Griffith JD: **Preferential nucleosome assembly at DNA triplet repeats from the myotonic dystrophy gene**. *Science (New York, NY* 1994, **265**(5172):669-671.

10. Cao H, Widlund HR, Simonsson T, Kubista M: **TGGA repeats impair nucleosome formation**. *J Mol Biol* 1998, **281**(2):253-260.
11. Wang YH, Gellibolian R, Shimizu M, Wells RD, Griffith J: **Long CCG triplet repeat blocks exclude nucleosomes: a possible mechanism for the nature of fragile sites in chromosomes**. *J Mol Biol* 1996, **263**(4):511-516.

Supplementary Figure Legends:

Figure S1. Same as the bottom panel of Figure 1, with rows labelled.

Figure S2. Same as Figure 1, except weights are shown after removing the unit-normalization of the inputs.

Figure S3. Same as the bottom panel of Figure S2 but with rows labelled.

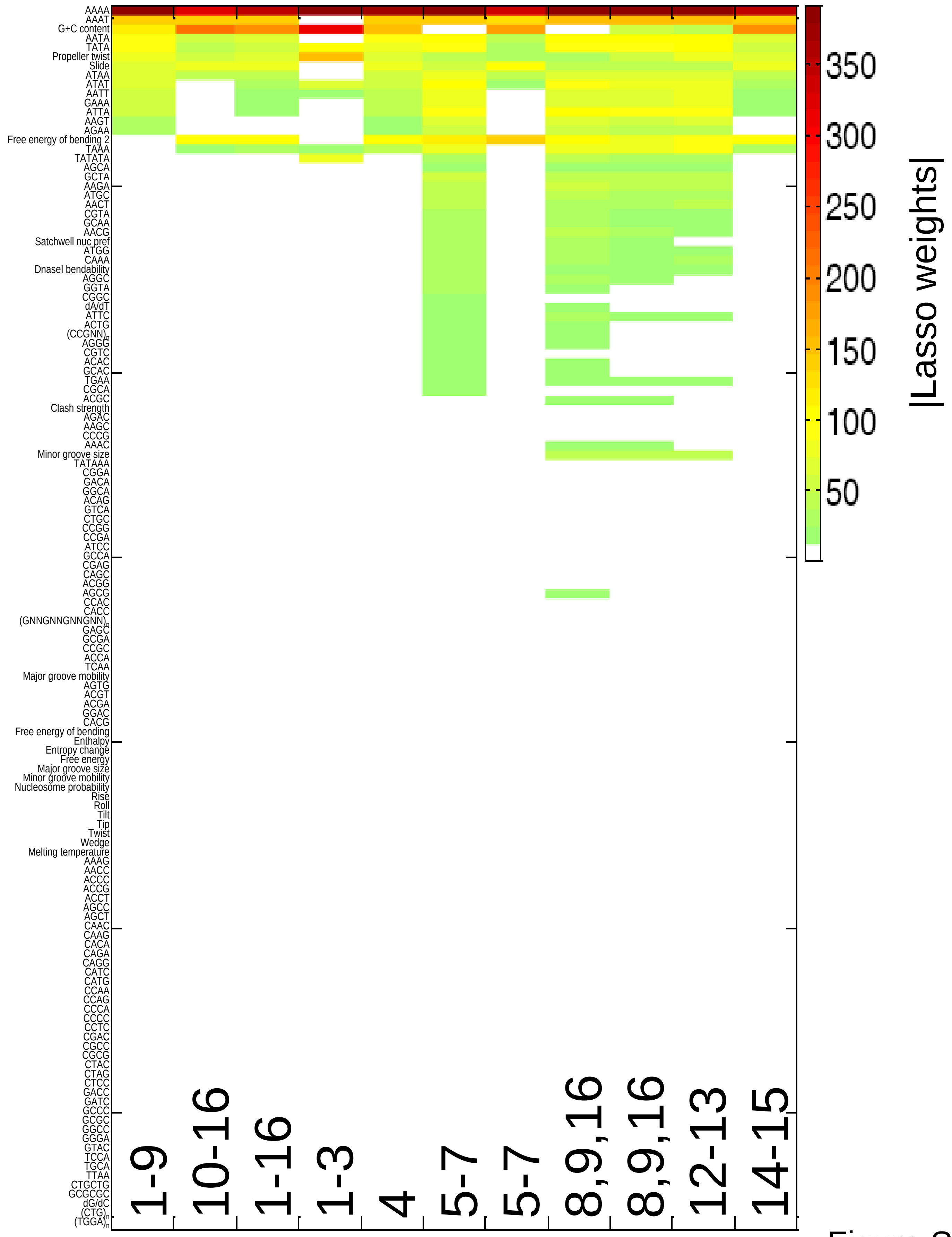
AAAA
AAAT
G+C content
AATA
TATA
Propeller twist
Slide
ATAA
ATAT
AATT
GAAA
ATTA
AAGT
AGAA
Free energy of bending 2
TAAA
TATATA
AGCA
GCTA
AAGA
ATGC
AACT
CGTA
GCAA
AACG
Satchwell nuc pref
ATGG
CAAA
DnaseI bendability
AGGC
GGTA
CGGC
dA/dT
ATTC
ACTG
(CCGNN)n
AGGG
CGTC
ACAC
GCAC
TGAA
CGCA
ACGC
Clash strength
AGAC
AAGC
CCCG
AAAC
Minor groove size
TATAAA
CGGA
GACA
GGCA
ACAG
GTCA
CTGC
CCGG
CCGA
ATCC
GCCA
CGAG
CAGC
ACGG
AGCG
CCAC
CACC
(GNNGNNGNNGNN)n
GAGC
GCGA
CCGC
ACCA
TCAA
Major groove mobility
AGTG
ACGT
ACGA
GGAC
CACG
Free energy of bending
Enthalpy
Entropy change
Free energy
Major groove size
Minor groove mobility
Nucleosome probability
Rise
Roll
Tilt
Tip
Twist
Wedge
Melting temperature
AAAG
AACC
ACCC
ACCG
ACCT
AGCC
AGCT
CAAC
CAAG
CACA
CAGA
CAGG
CATC
CATG
CCAA
CCAG
CCCA
CCCC
CCTC
CGAC
CGCC
CGCG
CTAC
CTAG
CTCC
GACC
GATC
GCCC
GCGC
GGCC
GGGA
GTAC
TCCA
TGCA
TTAA
CTGCTG
GCGCGC
dG/dC
(CTG)n
(TGGA)n
1-9
10-16
1-16
1-3
4
5-7
5-7
8,9,16
8,9,16
12-13
14-15
350
300
250
200
150
100
50
|Lasso weights|

Figure S1

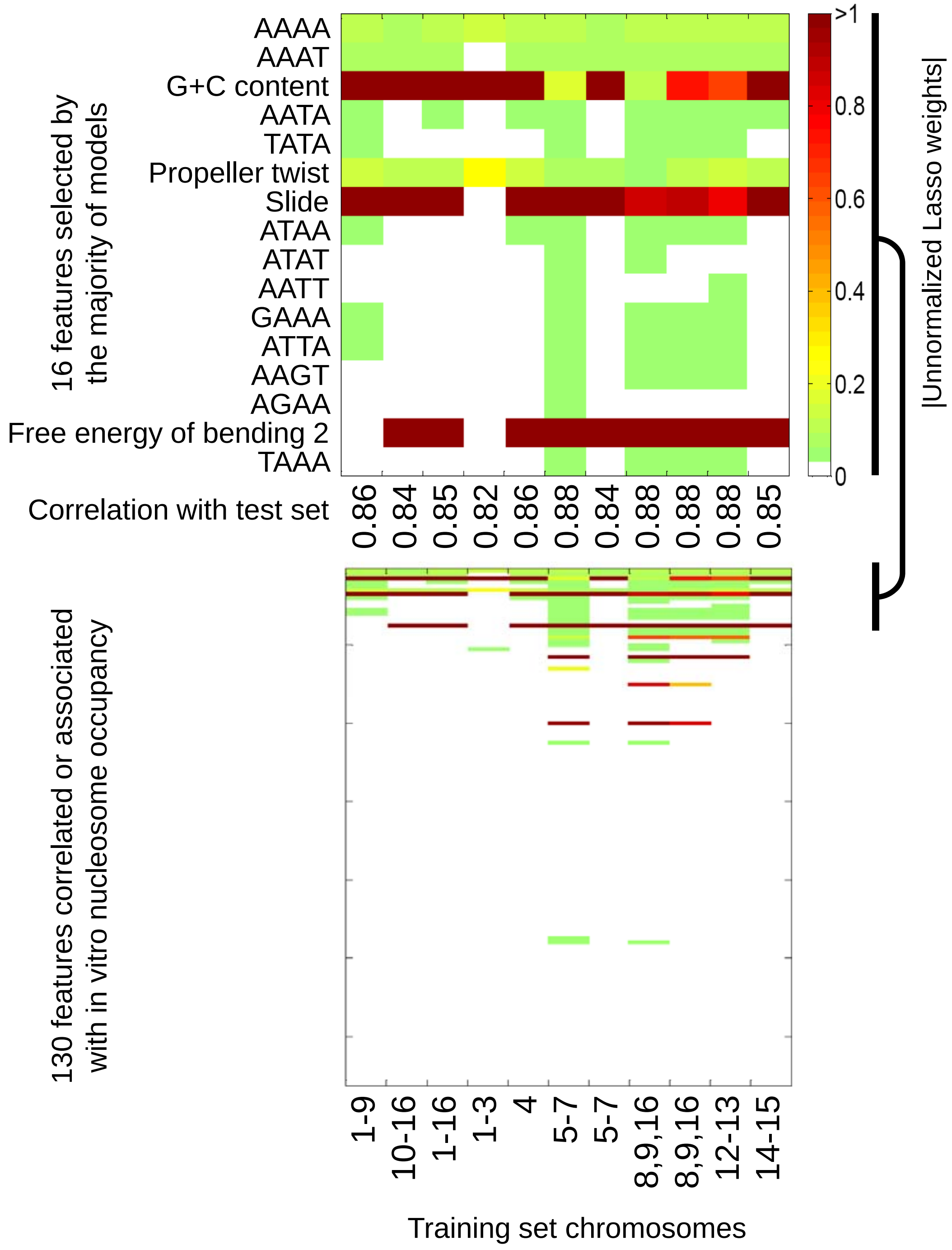

16 features selected by the majority of models
AAAA
AAAT
G+C content
AATA
TATA
Propeller twist
Slide
ATAA
ATAT
AATT
GAAA
ATTA
AAGT
AGAA
Free energy of bending 2
TAAA
Correlation with test set
0.86 0.84 0.85 0.82 0.86 0.88 0.84 0.88 0.88 0.88 0.85
>1
0.8
0.6
0.4
0.2
0
|Unnormalized Lasso weights|
130 features correlated or associated with in vitro nucleosome occupancy
1-9 10-16 1-16 1-3 4 5-7 5-7 8,9,16 8,9,16 12-13 14-15
Training set chromosomes

Figure S2

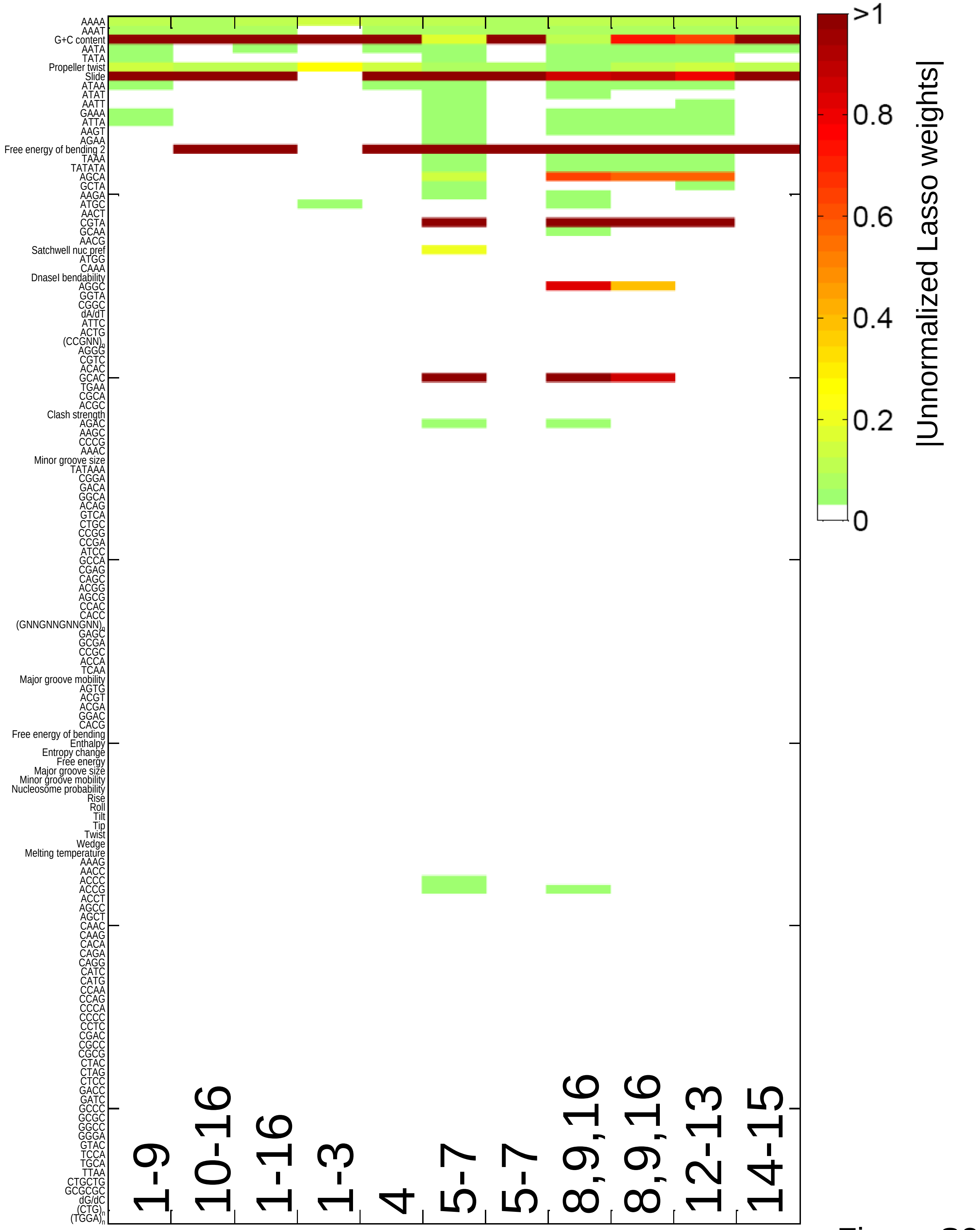
AAAA
AAAT
G+C content
AATA
TATA
Propeller twist
Slide
ATAA
ATAT
AATT
GAAA
ATTA
AAGT
AGAA
Free energy of bending 2
TAAA
TATATA
AGCA
GCTA
AAGA
ATGC
AACT
CGTA
GCAA
AACG
Satchwell nuc pref
ATGG
CAAA
DnaseI bendability
AGGC
GGTA
CGGC
dA/dT
ATTC
ACTG
(CCGNN)$_n$
AGGG
CGTC
ACAC
GCAC
TGAA
CGCA
ACGC
Clash strength
AGAC
AAGC
CCCG
AAAC
Minor groove size
TATAAA
CGGA
GACA
GGCA
ACAG
GTCA
CTGC
CCGG
CCGA
ATCC
GCCA
CGAG
CAGC
ACGG
AGCG
CCAC
CACC
(GNNGNNGNNGNN)$_n$
GAGC
GCGA
CCGC
ACCA
TCAA
Major groove mobility
AGTG
ACGT
ACGA
GGAC
CACG
Free energy of bending
Enthalpy
Entropy change
Free energy
Major groove size
Minor groove mobility
Nucleosome probability
Rise
Roll
Tilt
Tip
Twist
Wedge
Melting temperature
AAAG
AACC
ACCC
ACCG
ACCT
AGCC
AGCT
CAAC
CAAG
CACA
CAGA
CAGG
CATC
CATG
CCAA
CCAG
CCCA
CCCC
CCTC
CGAC
CGCC
CGCG
CTAC
CTAG
CTCC
GACC
GATC
GCCC
GCGC
GGCC
GGGA
GTAC
TCCA
TGCA
TTAA
CTGCTG
GCGCGC
dG/dC
(CTG)$_n$
(TGGA)$_n$
1-9
10-16
1-16
1-3
4
5-7
5-7
8,9,16
8,9,16
12-13
14-15
>1
0.8
0.6
0.4
0.2
0
|Unnormalized Lasso weights|

Figure S3
